# Serum selenium levels and prostate cancer risk: A MOOSE-compliant meta-analysis

**DOI:** 10.1097/MD.0000000000005944

**Published:** 2017-02-03

**Authors:** Zhigang Cui, Dezhong Liu, Chun Liu, Gang Liu

**Affiliations:** a Department of Urology, The 306th Hospital of Chinese PLA; b Department of Urology, The General Hospital of Chinese PLA Rocket Force, Beijing, China.

**Keywords:** meta-analysis, prostate cancer, selenium, serum

## Abstract

Some observational studies have shown that elevated serum selenium levels are associated with reduced prostate cancer risk; however, not all published studies support these results. A literature search of PubMed, Embase, Medline, and the Cochrane Library up until September 2016 identified 17 studies suitable for further investigation. A meta-analysis was conducted on these studies to investigate the association between serum selenium levels and subsequent prostate cancer risk. Pooled odds ratios (ORs) and 95% confidence intervals (CIs) were used to evaluate the overall OR of prostate cancer for the highest versus the lowest levels of serum selenium. We found a pooled OR (95% CI) of 0.76 (0.64, 0.91; *P* < 0.05). In subgroup analysis, an inverse association between serum selenium levels and prostate cancer risk was found in each of case–control studies, current and former smokers, high-grade cancer cases, advanced cancer cases, and different populations. Such correlations were not found for subgroups containing each of cohort studies, nonsmokers, low-grade cancer cases, and early stage cancer cases. In conclusion, our study suggests an inverse relationship between serum selenium levels and prostate cancer risk. However, further cohort studies and randomized control trials based on non-Western populations are required.

## 1 Introduction

Prostate cancer is a common cancer in men, accounting for approximately 25% of all cancers, and has the second highest incidence of cancer in men worldwide.^[1]^ In 2015, more than one million new prostate cancer patients were diagnosed, presenting a tremendous burden for public health.^[2]^ Although much effort has been directed toward prostate cancer prevention, many aspects of its etiology are still unknown. To address this serious challenge, it is necessary to explore strategies that might reduce the incidence of prostate cancer.

Research has suggested that trace elements play an important role in the biological processes underlying prostate cancer. Selenium, an essential component of antioxidants, has attracted much attention,^[3,4]^ and oxidative stress has been shown to be associated with the pathogenesis of prostate cancer.^[5]^ Excess production of free radicals or a deficiency in antioxidant defences can cause elevated oxidative stress, which in turn leads to cytoarchitecture and DNA damage.^[6]^ However, selenium can mitigate such damage by reducing free radicals.^[7]^ Selenium plays an important role in reproduction, acts as an antioxidant, and has antiaging activities. It is also implicated in many degenerative conditions, including inflammation, neurological disease, and cancer.^[8]^

A 1969 study suggested that cancer mortality in the United States was inversely correlated with the geographic distribution of selenium in the soil.^[9]^ This was the first report to suggest that selenium deficiency might be related to cancer. Subsequent studies have consistently concluded that increased serum selenium levels are associated with a reduced risk of prostate cancer.^[10–12]^ However, other observational studies have not found an inverse relationship between serum selenium levels and prostate cancer risk.^[13–15]^ These inconsistent results may be partly because of differences in population, study design, smoking status, and other confounding characteristics of participants. Moreover, sample sizes were relatively small in each study, limiting the strength of evidence. To increase statistical power and clarify these conflicting results, a meta-analysis was conducted to assess the association between serum selenium levels and the risk of developing prostate cancer.

## 2 Materials and methods

### 2.1 Study identification

A database search was performed using PubMed, Embase, Medline, and the Cochrane Library and included literature up until September 2016. The search terms “serum selenium” or “plasma selenium,” and “prostate carcinoma” or “prostate cancer” were used either as a combination of free text or as medical subject heading (MeSH) terms. Additionally, further studies were identified within the reference lists of retrieved articles.

### 2.2 Inclusion criteria

The following criteria were selected for inclusion of a study in the meta-analysis: the study reported the association between serum selenium levels and prostate cancer risk by comparing the risk for subjects with the highest serum selenium levels against the risk for subjects with the lowest serum selenium levels; the study had a cohort, case–control, or randomized controlled trial (RCT) design; the publication language was English; and where there were duplicate publications using the same study population or by the same authors, we included only the most recent publication.

### 2.3 Assessment of study quality

RCTs were assessed for quality using the Cochrane Collaboration's “Risk of bias” tool. Cohort and case–control studies were assessed according to the primary criteria for nonrandomized and observational studies for meta-analyses, using the Newcastle-Ottawa Quality Assessment scale.

### 2.4 Data collection

For each study, the following data were collected in a standardized data extraction form: first author's last name, year of publication, study design, study population, range of ages of subjects, sample size, odds ratio (OR) and 95% confidence interval (CI) of prostate cancer risk from the most fully adjusted model for the highest against the lowest levels of serum selenium, and quality scores.

This study was a meta-analysis; as such, ethical approval was not required. All analyses were conducted by 2 independent authors.

### 2.5 Statistical analysis

In the present study, statistical analyses were performed using STATA 12.0. The combined OR and corresponding 95% CI were used to estimate the relationship between serum selenium levels and prostate cancer risk. *Q* and *I*^*2*^ statistics were used to assess heterogeneity across the included studies.^[16]^ Subgroup analysis was performed to identify the effect of different factors on the overall risk assessment. A sensitivity analysis was also conducted to investigate the influence of individual studies on the overall risk estimate. Publication bias was measured using a funnel plot and Egger test.^[17,18]^ Differences with *P* values <0.05 were considered to be statistically significant.

## 3 Results

### 3.1 Study selection and characteristics

**Figure 1:**
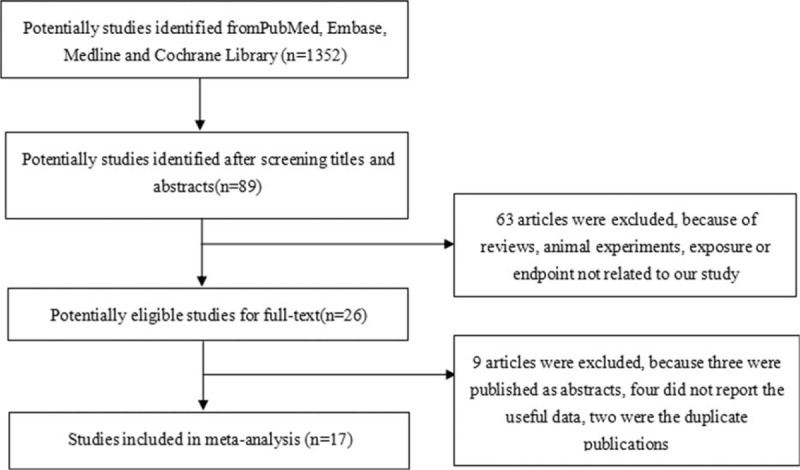
The process of study selection.

According to the search criteria, we indentified 1352 articles for further investigation. However, most were excluded after reviewing titles, abstracts, or full-text, either because they were not relevant to our study, they were review articles, or there was some other reason. A total of 17 studies were selected for inclusion in our meta-analysis, within which there were 6136 prostate cancer cases and >34,901 controls or participants.^[10–15,19–29]^Figure 1 presents the study selection process.

Among the 17 included studies, 12 were case–control,^[10–14,19,20,22,23,25,26,28]^ 4 were cohort studies,^[15,24,27,29]^ and 1 was a RCT.^[21]^ Ten studies were conducted using American populations,^[11–13,19–25]^ 1 Danish,^[29]^ 2 Swedish,^[10,15]^ 1 Iranian,^[26]^ 1 Finnish,^[27]^ and 2 were conducted using a mixed population from several European countries.^[14,28]^ All ORs and corresponding 95% CIs were calculated by comparing the risk of prostate cancer associated with highest serum selenium levels against the risk associated with the lowest serum selenium levels. Table 1 presents the main characteristics of the included studies.^[10–15,19–29]^

**Table 1:**
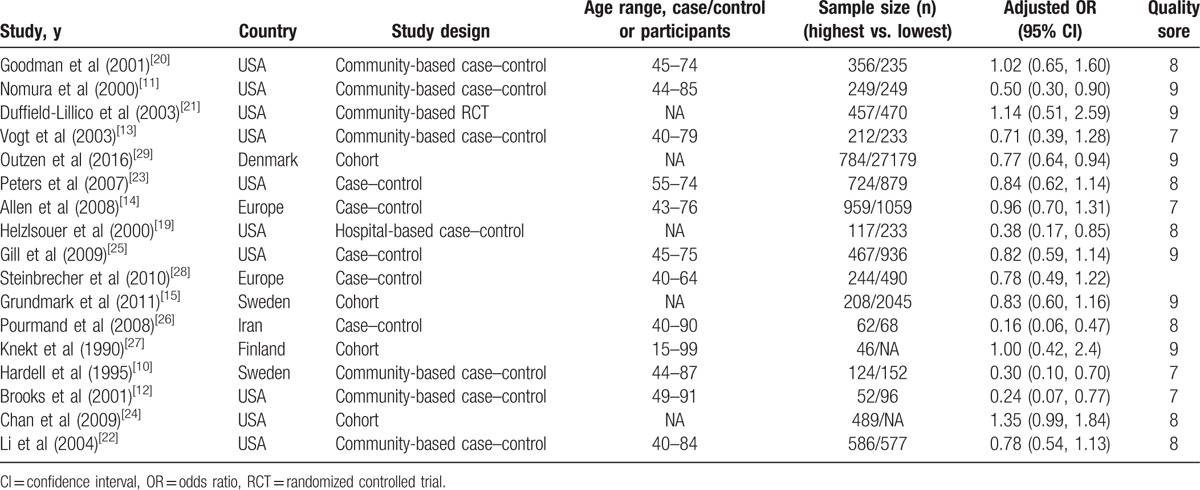
The characteristics of included studies.

### 3.2 Main analysis

**Figure 2:**
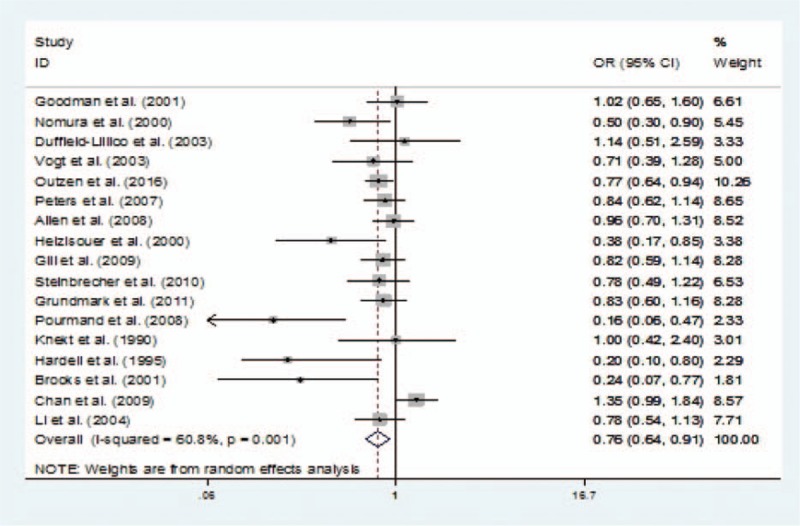
Meta-analysis of the relationship between serum selenium levels and subsequent all prostate cancer risks.

Figure 2 presents the association between serum selenium levels and prostate cancer risk for the highest against the lowest levels of serum selenium. Among the included studies, 6 showed an inverse association between serum selenium levels and prostate cancer risk.^[10–12,19,26,28]^ However, the results showed that there was an inverse relationship between serum selenium levels and prostate cancer risk. In other words, the population with higher serum selenium levels may have a lower subsequent prostate cancer risk. The pooled OR (95% CI) from all the studies was 0.76 (0.64, 0.91); however, we also found significant evidence of heterogeneity (*I*^*2*^ = 60.8%, *P* = 0.001).

### 3.3 Sensitivity analysis

**Figure 3:**
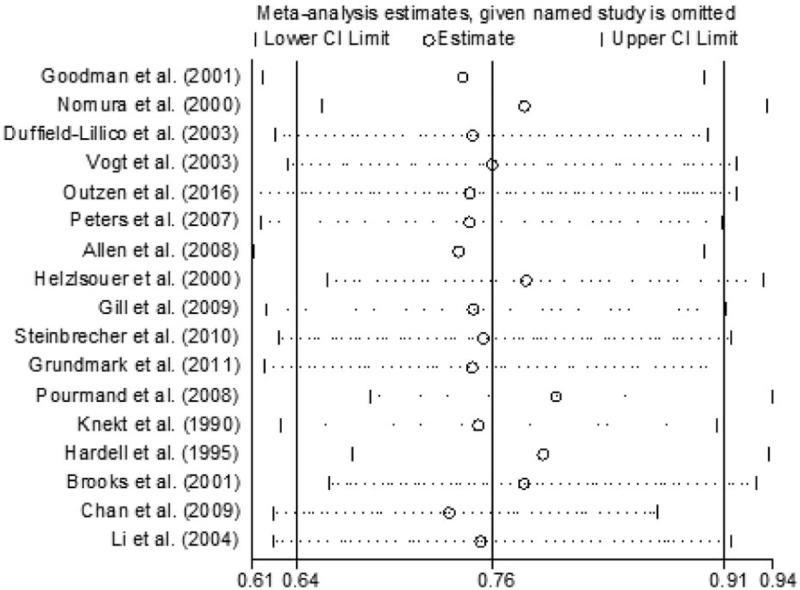
Forest plot for sensitivity analysis.

Removing 1 study at a time, the combined OR remained similar (Fig. 3); therefore, no single study significantly altered the overall OR.

### 3.4 Publication bias

**Figure 4:**
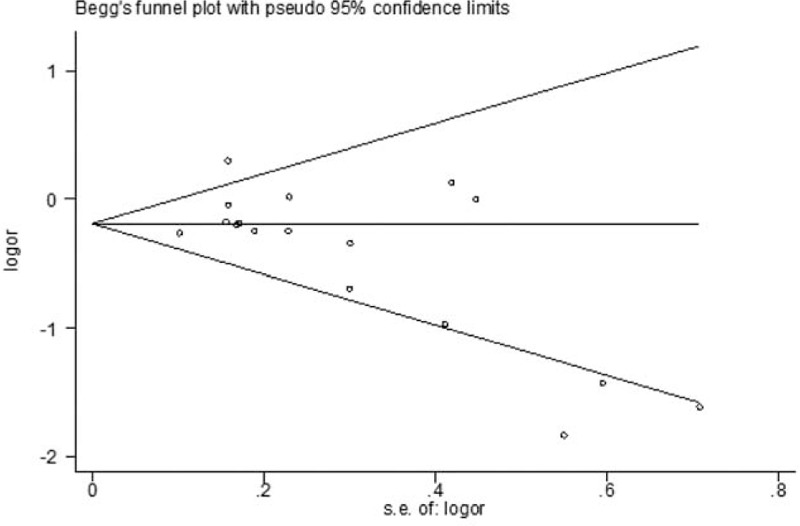
Forest plot for publication bias.

The funnel plot and Egger regression test (Fig. 4) showed that there was no evidence of publication bias in the present study (*P* = 0.127).

### 3.5 Subgroup-analysis

Lastly, we conducted a subgroup analysis to assess the influences of study design, study population, and smoking status on the estimation of overall OR (Table 2). Individually, we found inverse relationships between serum selenium levels and prostate cancer risk in the subgroups of case-control studies, current smokers and former smokers. Moreover, these inverse relationships were not altered by population distributions. No specific relationships were identified between serum selenium levels and prostate cancer risk in the subgroups of cohort studies or nonsmokers.

**Table 2:**
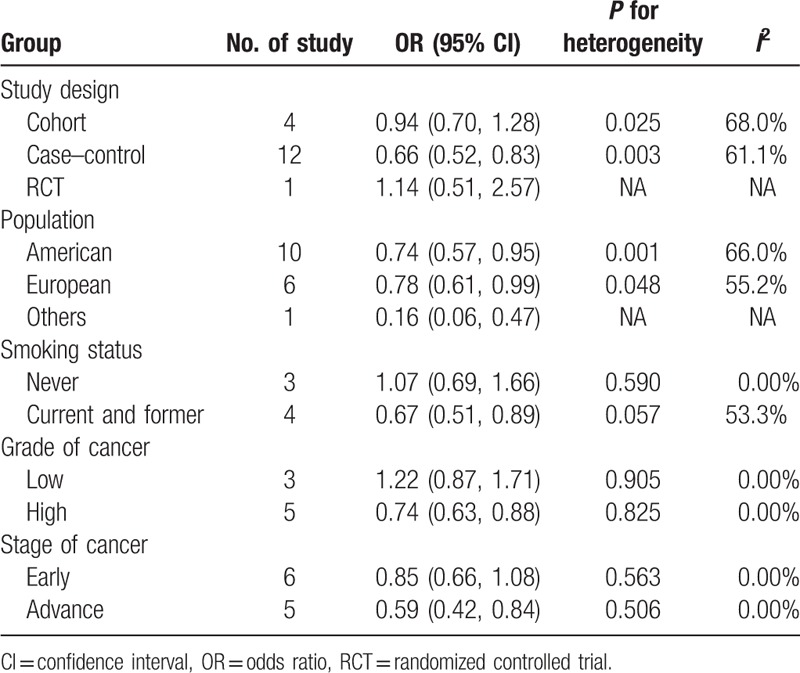
The results of subgroup analyses were based on study design, population, smoking status, grade, and stage of cancer.

## 4 Discussion

Our study shows that increased serum selenium is associated with a reduced risk of prostate cancer. Selenium is an antioxidant and anticarcinogen.^[30]^ It is also an important component of 3 major proteins: selenoprotein-P, glutathione peroxidase (GPx), and albumin. Within GPx, selenium is part of the most important antioxidant enzyme system that prevents cellular injury.^[31]^ In addition, selenium inhibits the expression of some oncogenes and promotes apoptosis.^[32]^ Selenium is also part of the most important antioxidant enzyme system involved in preventing peroxidation of cells.^[33]^ Like vitamin E, selenium may be a superior scavenger of reactive nitrogen oxide species, and has the capacity to decrease inflammation.^[33]^ Selenium also inhibits cell proliferation and decreases cell cycle progression through the reduction of cyclin in prostate cancer cell lines.^[34]^

The relationship between serum selenium and the risk of prostate cancer has been studied for many years. In 1990, a cohort study was conducted by Knekt et al that showed that a elevated serum selenium level was not associated with an increased prostate cancer risk^[10]^; however, several studies presented conflicting results.^[11,12,19]^ More recent studies have presented results that have supported those of Knekt et al. We hypothesized that the inconsistent conclusions from previous studies might be because of differences in study area, study design, sample size, follow-up time, age range of population, and quality of study. Therefore, we thought it timely to carry out a new meta-analysis of the data from these studies.

Our study showed an inverse relationship between serum selenium levels and prostate cancer risk. This result was inconsistent with a previous study that assessed the relationship between selenium supplementation and prostate cancer risk.^[35]^ However, the result of that study was unsurprising, given that serum selenium levels reflect long-term accumulation of selenium in the human body and selenium supplements may not have been adequately absorbed by the body. Moreover, the sample size was much larger in our study, suggesting a more credible result.

Despite the large sample size from the combined studies in our meta-analysis, there was strong evidence of heterogeneity. Even though we could not identify any clear sources of the heterogeneity, differences in the populations, study design, smoking status, grade and stage of cancer, and other varied characteristics of participants may at least partially explain this result.

An inverse relationship between serum selenium levels and prostate cancer risk was found in the case–control studies but not in the cohort studies. This result was unsurprising because there were only a small number of studies in the cohort group. Moreover, most of these cohort studies did not find an inverse relationship to begin with.

We also observed an inverse association between serum selenium and prostate cancer risk in smokers, but not in nonsmokers. Smoking is a risk factor for the incidence of prostate cancer^[36]^; however, many studies have suggested that other antioxidants, such as vitamin E, are protective against prostate cancer, particularly in smokers.^[37]^ Other studies have shown that the antioxidant effects of selenium observed in smokers may be enhanced by the presence of oxidative response elements in the promoter regions of genes encoding selenoenzymes.^[38]^

There were several strengths to our study. First, it was comprehensive in evaluating the association of serum selenium levels and prostate cancer risk because it included all relevant studies. Second, our study had a large sample size, thereby enhancing the statistical power and increasing the reliability of the results. Most importantly, we found a significantly inverse relationship between serum selenium levels and prostate cancer risk. Because serum selenium is a broad indicator of selenium in the diet, our study suggests that increased selenium intake might help prevent the development of prostate cancer.

The present meta-analysis has several limitations. First, we only evaluated serum selenium levels. The serum selenium reflects long-term selenium intake and is relatively accurate in ranking selenium intake in population studies.^[39]^ Our study supports the hypothesis that there is a relationship between serum selenium levels and prostate cancer risk in populations with long-term steady selenium intake. However, selenium measurements in toenail samples and other biomaterials are needed to evaluate short-term and transient changes in selenium intake. Second, there were residual confounders and unmeasured factors present in each of the included studies. Adjustments were made for these factors within each study, but the adjustments were not completely consistent between studies. Residual confounders may alter the measured effect of selenium on the risk of cancer. For example, men with long-term vitamin C supplementation have a 21% reduced risk of prostate cancer.^[40]^ Third, there was strong evidence of heterogeneity among the included studies. Although differences in populations, study design, smoking status, grade, and stage of cancer may explain this heterogeneity, other differences present in the studies should also be considered. For example, there may be measurement errors arising from the use of different methods, and facilities and staff involved in the studies may bias estimates of effect. Fourth, although cohort studies and RCTs are generally considered best for investigating effect, few such studies have been conducted to evaluate the association between serum selenium levels and prostate cancer risk. Finally, the studies included in our analysis primarily investigated Western populations, in particular Americans and Europeans. Therefore, further studies investigating other populations should be carried out in the future.

## 5 Conclusions

Our meta-analysis suggests that there is an inverse relationship between serum selenium levels and subsequent prostate cancer risk. Further investigations using well-designed cohort studies and RCTs based on non-Western populations are required.
